# Disruption of *N*‐acyl‐homoserine lactone‐specific signalling and virulence in clinical pathogens by marine sponge bacteria

**DOI:** 10.1111/1751-7915.12867

**Published:** 2017-11-03

**Authors:** José A. Gutiérrez‐Barranquero, F. Jerry Reen, María L. Parages, Ronan McCarthy, Alan D. W. Dobson, Fergal O'Gara

**Affiliations:** ^1^ BIOMERIT Research Centre School of Microbiology University College Cork National University of Ireland Cork Ireland; ^2^ School of Microbiology University College Cork National University of Ireland Cork Ireland; ^3^ Human Microbiome Programme School of Biomedical Sciences Curtin Health Innovation Research Institute Curtin University Perth WA Australia; ^4^ Curtin Health Innovation Research Institute (CHIRI) Curtin University Perth WA Australia; ^5^ School of Biomedical Sciences Faculty of Health Sciences Curtin University Perth WA Australia; ^6^ Present address: Instituto de Hortofruticultura Subtropical y Mediterránea La Mayora Departamento de Microbiología Facultad de Ciencias Universidad de Málaga 29071 Málaga Spain; ^7^ Present address: Departamento de Ecología Facultad de Ciencias Universidad de Málaga 29071 Málaga Spain

## Abstract

In recent years, the marine environment has been the subject of increasing attention from biotechnological and pharmaceutical industries. A combination of unique physicochemical properties and spatial niche‐specific substrates, in wide‐ranging and extreme habitats, underscores the potential of the marine environment to deliver on functionally novel bioactivities. One such area of ongoing research is the discovery of compounds that interfere with the cell–cell signalling process called quorum sensing (QS). Described as the next generation of antimicrobials, these compounds can target virulence and persistence of clinically relevant pathogens, independent of any growth‐limiting effects. Marine sponges are a rich source of microbial diversity, with dynamic populations in a symbiotic relationship. In this study, we have harnessed the QS inhibition (QSI) potential of marine sponge microbiota and through culture‐based discovery have uncovered small molecule signal mimics that neutralize virulence phenotypes in clinical pathogens. This study describes for the first time a marine sponge *Psychrobacter* sp. isolate B98C22 that blocks QS signalling, while also reporting dual QS/QSI activity in the *Pseudoalteromonas* sp. J10 and *Paracoccus* JM45. Isolation of novel QSI activities has significant potential for future therapeutic development, of particular relevance in the light of the pending perfect storm of antibiotic resistance meeting antibiotic drug discovery decline.

## Introduction

The spectre of a post‐antibiotic era is emerging as a very real and imminent threat as the discovery void in antibiotic development is met with an ongoing increase in antibiotic resistance among bacterial and fungal pathogens alike. The rise of antibiotic resistance has become one of the major global health issues of recent years (Fernández and Hancock, 2012; Tanwar *et al*., 2014), as the report in 2014 of the World Health Organization (WHO) describes (http://www.who.int/mediacentre/news/releases/2014/amr-report/en/). Therefore, there is an urgent need for new strategies to combat microbial diseases, which are more effective and less susceptible in generating of resistance as conventional antibiotics. One strategy is to target virulence systems in the pathogen, neutralizing key virulence factors and in some cases the basis for resistance and tolerance to antibiotics (Cegelski *et al*., 2008; Cooper and Shlaes, 2011; LaSarre and Federle, 2013). In this context, biofilm formation and strategies to counteract it have received much attention in recent years.

Biofilm formation is one of the major virulence factors associated with clinical human pathogens, and this mode of growth has been described to be directly involved with an increase in antibiotic resistance (Hoiby *et al*., 2011). As with many multicellular processes, biofilm formation is known to be controlled by a process called quorum sensing (QS). QS is a bacterial cell–cell communication signalling system that controls gene expression in response to changes in cell density. As part of this system, autoinducer molecules enable bacteria to coordinate and control behaviours in a synchronized way (Papenfort and Bassler, 2016). The signalling components of QS systems are different between Gram‐negative and Gram‐positive bacteria, primarily with respect to the autoinducer signalling molecules and their respective receptors. Although several distinct autoinducing systems have been described in Gram‐negative bacteria, by far, the most widely distributed are the *N*‐acyl‐homoserine lactones (AHLs) (Ng and Bassler, 2009; Schertzer *et al*., 2009). AHL–QS signalling is a key component of the biofilm mode of growth in many pathogens. It has not been shown to be required for essential cellular processes (Rasmussen and Givskov, 2006). While this was initially thought to support the claim that the selective pressure associated with conventional antibiotic use would not generally apply, recent studies have described the selection of QS mutants (Maeda *et al*., 2012; García‐Contreras *et al*., 2016). Furthermore, an adaptable QS circuitry has been described in variants from patients with Cystic Fibrosis, suggesting that circuit heterogeneity may apply for many target pathogens (Feltner *et al*., 2016). Notwithstanding this, molecules that are able to inhibit the QS system (quorum sensing inhibition molecules, QSIm), and by extension biofilm formation or other related virulence phenotypes, remain promising targets to fight opportunistic human pathogens (Rasmussen and Givskov, 2006; Atkinson and Williams, 2009; Njoroge and Sperandio, 2009; Kalia, 2013).

Targeting AHL‐based signalling systems has already been shown to be an effective anti‐biofilm strategy with compounds such as halogenated furanones, coumarin and enzymes such as acylases and lactonases, which have all been shown to have anti‐pathogenic activity (Dong *et al*., 2001, 2002; Hentzer *et al*., 2003; Park *et al*., 2005; O'Loughlin *et al*., 2013; Gutierrez‐Barranquero *et al*., 2015). AHL signalling is based on structure‐specific interactions between small molecular signals and their cognate receptor proteins. AHLs can vary in chain length from C4 to C18, with modifications on the homoserine‐lactone framework (Churchill and Chen, 2011) resulting in a diverse collection of potential signals. In addition, a new QS system using aryl‐homoserine lactones as autoinducer molecules has been reported in *Rhodopseudomonas palustris* and in *Bradyrhizobium* (Schaefer *et al*., 2008;. Ahlgren *et al*., 2011).

The discovery of AHL–QS systems in marine Gram‐negative bacteria (Nealson *et al*., 1970; Ruby, 1996; Hastings and Greenberg, 1999) has led to a concerted focus on this relatively unique ecological niche for cell signalling mimics. The marine environment is emerging as a rich and untapped source of novel bioactive molecules with invaluable biotechnological and pharmaceutical potential (Reen *et al*., 2015a,b). Specifically, marine sponges and their symbiotic microbiomes have been described to be one of the major sources of novel bioactive molecules (Hardoim and Costa, 2014; Blunt *et al*., 2016). Different studies support the potential for use of culturable bacteria from marine sponges as new platforms for the discovery of novel anti‐pathogenic compounds, including those with a QSI mode of action (Skindersoe *et al*., 2008; Dobretsov *et al*., 2011; Pejin B *et al*., 2014; Mai *et al*., 2015; Saurav *et al*., 2016). Therefore, unravelling the QSI potential of marine microorganisms, and subsequently, linking their bioactive potential to the inhibition of QS‐regulated virulence phenotypes of bacterial pathogens is a key research and translational biodiscovery goal.

In this study, we have profiled the potential QSI capabilities from a bacterial collection isolated from marine sponges sourced from multiple geographical locations. A combined high‐throughput biosensor‐based screening protocol was designed, encompassing both generalized disruption and subsequently structure‐specific interference with QS. QSI activities were found to be non‐enzymatic (QSI by enzymes: quorum quenching, QQ) and exhibited a significant degree of structural specificity for disruption of C4, 3OC8, 3OC10 and 3OC12 AHL signalling molecules. This activity was extended to anti‐biofilm activity against both the nosocomial pathogen *P. aeruginosa* PA14 and the biofouling agent *Bacillus subtilis* CH8a. In addition, disruption of other important QS virulence phenotypes in *P. aeruginosa* PA14 was demonstrated. In this work, we have described for the first time the marine sponge *Psychrobacter* sp. B98C22 with QSI activity, and interestingly, another two marine sponge isolates which showed dual QS/QSI activity (*Pseudoalteromonas* sp. J10 and *Paracoccus* sp. JM45). The results in this study further highlight the potential of marine sponge bacteria as a valuable source of diverse QSI compounds that could play a vital role in controlling the new era of emergence of multidrug‐resistant pathogens.

## Results

### Identification and phylogenetic analysis of QSI‐producing marine sponge bacteria

**Figure 1:**
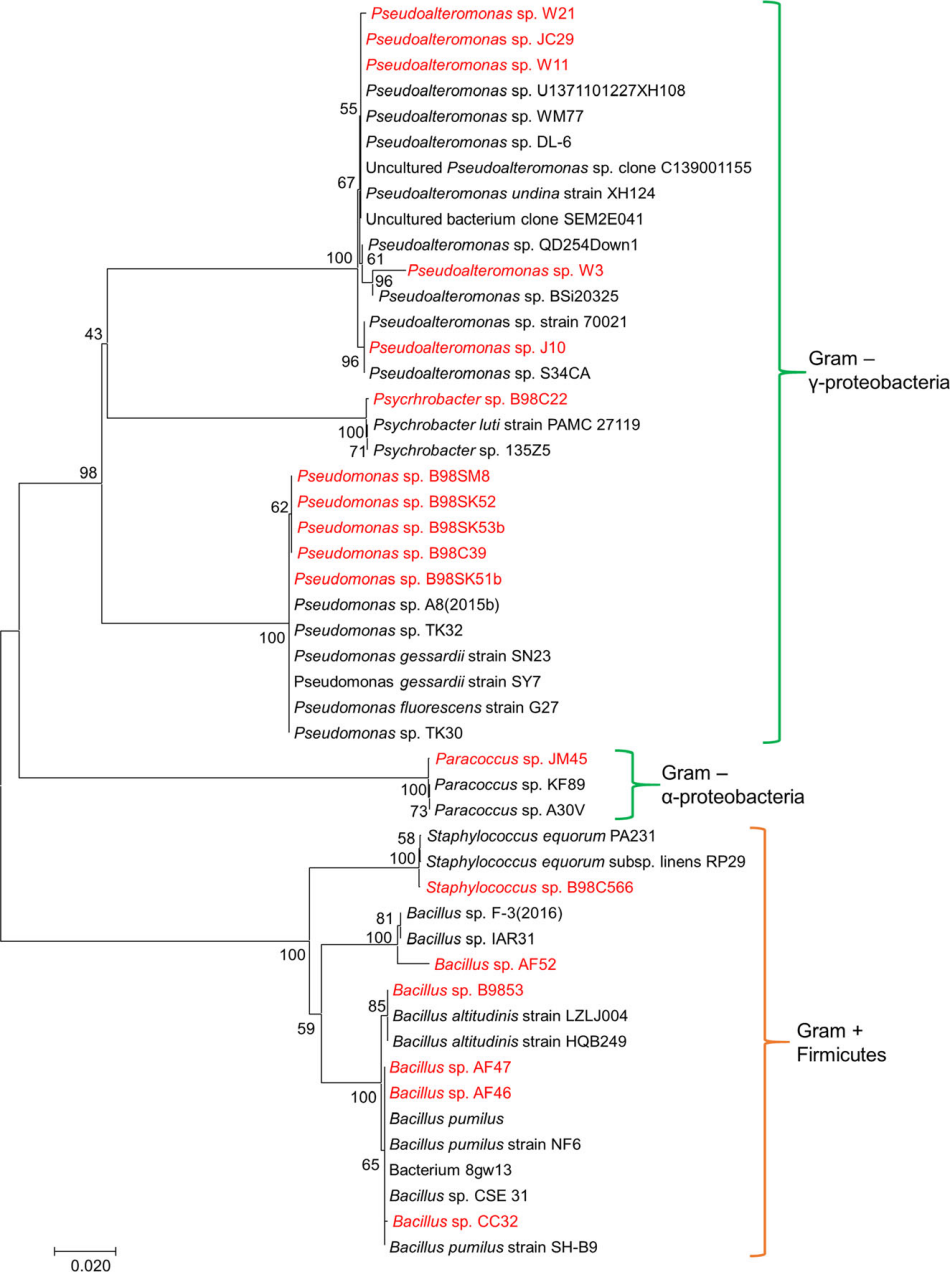
Phylogenetic distribution based on the 16S rRNA sequence of the QQ marine sponge bacteria. Isolates with QQ activity isolated from this study are highlighted in red. Gram‐negative bacteria from γ and α Proteobacteria classes are marked in green. Gram‐positive bacteria belonging to the Phylum Firmicutes, Bacilli Class, are marked in orange.

A screening and validation pipeline was designed to mine for QSI‐producing candidates from a collection of culturable bacteria isolated from a diverse array of marine sponge samples (Fig. S1). Three different biosensor reporter strains, *S. marcescens* SP15, *C. violaceum* DSM 30191 and *A. tumefaciens* NTL4 were used to detect QQ activity against short‐, medium‐ and long‐chain AHLs respectively. Analysis of a total of 440 bacterial isolates led to the identification of 18 isolates (4.1%) with the potential ability to inhibit the QS system of at least one biosensor reporter strain. After the initial screening, these 18 bacterial isolates were taxonomically identified by 16S rDNA sequencing, and a phylogenetic distribution of the QSI candidates was performed (Fig. 1). QSI isolates were identified as belonging primarily to the Gram‐negative Gammaproteobacteria class; five *Pseudomonas* sp. strains (B98C39, B98SK51b, B98SK53b, B98SK52 and B98SM8), five *Pseudoalteromonas* sp strains (J10, JC29, W3, W11 and W21) and one *Psychrobacter* sp. strain (B98C22). In addition, one strain belonging to the Alphaproteobacteria class (*Paracoccus* sp. JM45) was also identified. Furthermore, Gram‐positive QSI candidates belonging to the Phylum Firmicutes were also identified (five *Bacillus* sp. strains: AF46, AAF47, AF52, B9853 and CC32 and one *Staphylococcus* sp. strain: B98C566). A comparative analysis of QQ and QSI activities described in related bacteria to our study in previous reports could highlight the novelty of the activities uncovered in this work (Table 1).

**Table 1:** Comparative analysis of QQ/QSI activities from related bacteria to the novel activities from this study

Bacterial species	Source	Proposed activity	Phenotypes/Bioassay used	References
*Bacillus*	Different non‐sponge marine origins	Lactonase	Biosensors	(Romero *et al*., 2011)
*Bacillus* sp. QSI‐1	Fish gut	Lactonase	*C. violaceum* biosensor protease, haemolytic activity, biofilm	(Chu *et al*., 2014)
*B. cereus* D28	Marine sediment	Cyclic dipeptide	Biosensors, bioluminescence in *Vibrio*	(Teasdale *et al*., 2011)
*B. cereus*	Soil	Not analysed	*C. violaceum* biosensor, biofilm	(Wahman *et al*., 2015)
*Bacillus horikosshi*	Coral	Extracts	Biosensors	(Thenmozhi *et al*., 2009)
*Bacillus pumilus*	Marine sediment	Acylase	Biosensors, *P. aeruginosa* and *Serratia* virulence	(Nithya *et al*., 2010)
*Bacillus sonorensis*	Soya sauce	Not analysed	*C. violaceum* biosensor	(Yin *et al*., 2012a)
*B. thuringiensis*,* B. cereus* and *B. mycoides*	Soil	Lactonase	Biosensors	(Dong *et al*., 2002)
*Bacillus Pseudalteromonas Pseudomonas*	Brown algae	Not analysed	*Serratia* biosensor	(Kanagasabhapathy *et al*., 2009)
*Bacillus Pseudomonas*	Soil	Not analysed	*C. violaceum* biosensor	(Chong *et al*., 2012)
*Halobacillus salinus*	Seagrass	Phenethylamide	Biosensors bioluminescence in *Vibrio*	(Teasdale *et al*., 2009)
*P aeruginosa*	Marine sponge	Cyclic dipeptide	Not directly related with QSI	(Jayatilake *et al*., 1996)
*P. aeruginosa*	Clinical sample	Acylase	Biosensors, AHL inhibition, Elastase, Pyocyanin	(Sio *et al*., 2006)
*P aeruginosa*	Clinical sample	QuiP acylase	Control its own QS system	(Huang *et al*., 2006)
*P. aeruginosa*	Clinical sample	PvdQ acylase	Control its own QS system	(Bokhove *et al*., 2010)
*P. aeruginosa*	Clinical sample	Lactonase SsoPox	Pyocyanin, protease, biofilm	(Guendouze *et al*., 2017)
*Pseudomonas*	Rhizosphere	Not analysed	*C. violaceum* biosensor with OC6, biocontrol activity	(Alymanesh *et al*., 2016)
*Staphylococus*	Clinical sample	Small molecules ‘yayurea A, B’	Biosensors, Pyocyanin. Biofilm	(Chu *et al*., 2013)
*Staphylococcus*	Marine sponge	Not analysed	Not tested	(Saurav *et al*., 2016)
*Staphylococcus saprophyticus*	Marine source	Cyclic dipeptide	Biosensors	(Li *et al*., 2013)
*Staphylococcus saprophyticus*	Sewage	Not analysed	*C. violaceum* biosensor with OC6	(Chan *et al*., 2015)
*Paracoccus*	Marine sponge	Three putative small molecules – structure unknown	Biosensors, Antimicrobial activity, Pyocyanin, Biofilm	(Saurav *et al*., 2016)
*Pseudoalteromonas* JG1	Water to rear healthy turbot	Enzymes	Genomic data	(Yu *et al*., 2013)
*Pseudoalteromonas*	Marine eukaryotes	Not analysed	*C. violaceum* biosensor, biofilm	(Busetti *et al*., 2015)
*Pseudoalteromonas*	Marine and estuarine waters	Not analysed	*Serrattia* and *C. violaceum* biosensors, bioluminescence in *Vibrio*	(Linthorne *et al*., 2015)
*Pseudoalteromonas*	Surface of different marine Eukarya	Enzymes	*E. coli* biosensor for AHL and AI‐2, biofilm	(Weiland‐Bräuer *et al*., 2016)
*Alteromonas*	Different non‐sponge marine origins	Acylase	Biosensors	(Romero *et al*., 2011)

All QQ activities were subsequently validated on both Marine Agar (MA) and SYP supplemented with 1.5% (w/v) sea salt. The higher concentration of sea salt in  MA affected pigment production in the case of *S. marcescens* SP15 and *C. violaceum* DSM 30191. As a result, the response of the biosensors was less intense, although the inhibition results were comparable in both media (Table 2). *Bacillus* sp. strains (AF46, AF47, AF52 and CC32) showed the most remarkable QSI activity, being able to block all three biosensors reporter strains, and more specifically, 3OC10 and 3OC12 AHLs when *A. tumefaciens* NTL4 was used. In contrast, *Psychrobacter* sp. B98C22 and *Staphylococcus* sp. B98C566 displayed the lowest promiscuity with regards to QSI activity, only showing activity against *S. marcescens* SP15. In general, QSI active strains did not exhibit antibacterial activity against the biosensor strains. However, all five *Pseudomonas* strains along with *Pseudoalteromonas* JC29 affected the growth of *S. marcescens* SP15, while three *Pseudomonas* strains (B98SK53b, B98SK52 and B98SM8) inhibited growth of *C. violaceum* DSM 30191 (Table 2). Although none of the other QSI isolates affected growth of the biosensor strains and do not appear to produce antibacterial activity under the conditions tested, several *Bacillus* strains did suppress the growth of two fish pathogens and a *Staphylococcus aureus* strain (Table 3). In addition, two of the QSI isolates (*Pseudoalteromonas* sp. J10 and *Paracoccus* sp. JM45) also possessed QS activity, being capable of activating the *A. tumefaciens* NTL4 biosensor in response to the production of long AHLs (Fig. S2). To our knowledge, this is the first description of dual‐acting QS and QSI isolates from the marine sponge environment.

**Table 2:** Quorum quenching activity shown by marine sponge bacterial isolates

QQ Isolate	Genus	Isolated from sponge	Biosensor reporter strains			
			SP15	DSM 30191	NTL4‐(3OC10)^a^	NTL4‐(3OC12)^b^
AF46	*Bacillus* sp.	Genus *Amphilectus*	++^c^	+^d^	+	++
AF47	*Bacillus* sp.	Genus *Amphilectus*	++	+	+	++
AF52	*Bacillus* sp.	Genus *Amphilectus*	+	−/+^e^	+	+
B9853	*Bacillus* sp.	Class Hexactinellida	++	−/+	++	−f
B98C39	*Pseudomonas* sp.	Class Hexactinellida	IG^a^	+	−/+	+
B98C22	*Psychrobacter* sp.	Class Hexactinellida	+	−	−	−
B98C566	*Staphylococcus* sp.	Class Hexactinellida	+	−	−	−
B98SK51b	*Pseudomonas* sp.	Class Hexactinellida	IG	+	−/+	+
B98SK52	*Pseudomonas* sp.	Class Hexactinellida	IG	IG	−/+	−/+
B98SK53b	*Pseudomonas* sp.	Class Hexactinellida	IG	IG	−/+	−/+
B98SM8	*Pseudomonas* sp.	Class Hexactinellida	IG	IG	+	−/+
CC32	*Bacillus* sp.	Genus *Cliona*	++	−/+	++	++
J10	*Pseudoalteromonas* sp.	Genus *Polymastia*	+	+	+	+
JC29	*Pseudoalteromonas* sp.	Genus *Polymastia*	IG	+	−	−
JM45	*Paracoccus* sp.	Genus *Polymastia*	−/+	−/+	+	+
W3	*Pseudoalteromonas* sp.	Genus *Axinella*	+	++	−/+	−/+
W11	*Pseudoalteromonas* sp.	Genus *Axinella*	+	+	−/+	−/+
W21	*Pseudoalteromonas* sp.	Genus *Axinella*	+	+	−/+	−/+

**a.** 3OC10: *N*‐(3‐Oxodecanoyl)‐l‐homoserine lactone.

**b.** 3OC12: *N*‐(3‐Oxododecanoyl)‐l‐homoserine lactone.

**c.** ++: strong pigment inhibition, QQ activity. Inhibition halo > 20.0 mm.

**d.** +: pigment inhibition, QQ activity. Inhibition halo from ˃ 2.5 mm to ≤ 20.0 mm.

**e.** −/+: weak response of pigment inhibition. Inhibition halo from 1.0 mm to ≤ 2.5 mm.

**f.** −: no inhibition of pigment, no QQ activity.

a IG: inhibition of growth.

**Table 3:** Antimicrobial activity of marine *Bacillus* sp. strains

Bacterial strains	Antimicrobial activity		
	Fish pathogens		Opportunistic human pathogen
	*V. anguillarum*	*E. tarda*	*S. aureus* NCDO949
*Bacillus*			
AF46	+^a^	−c	+
AF47	+	−	+
AF52	+	−	−
B9853	−/+^b^	−	+
CC32	+	−/+	+

**a.** +: antimicrobial activity. Growth inhibition halo from ˃ 2.5 mm to ≤ 20.0 mm.

**b.** −/+: weak response of antimicrobial activity. Growth inhibition halo from 1.0 mm to ≤ 2.5 mm.

**c.** −: no antimicrobial activity.

### Specific AHL inhibition by marine bacterial supernatants is not related to extracellular enzymatic activity

The role of AHLs in controlling adaptive behaviour in bacteria has been long established, with many virulence phenotypes displayed by bacterial pathogens under tight regulation by QS systems (Rutherford and Bassler, 2012). These systems are hierarchical and temporal in nature, with interconnecting downstream regulatory pathways the norm in many well‐studied bacterial pathogens. In order to decipher whether there was a structural specificity to the AHL inhibition activity of the different marine bacterial strains, the QS biosensor reporter assay was modified by adding specific exogenous AHLs. *S. marcescens* SP19 was used to detect structure‐specific inhibition against C4‐HSL, and *C. violaceum* CV026 was used to detect specific inhibition against C6‐HSL and 3OC8‐HSL. Six QSI strains (*Bacillus* sp. B9853, *Paracoccus* sp. JM45, *Psychrobacter* sp. B98C22, *Staphylococcus* sp. B98C566, *Pseudomonas* sp. B98SM8 and *Pseudoalteromonas* sp. J10) were able to inhibit activity towards a single AHL, namely 3OC8 (Table 4). Most of the marine strains (*Bacillus* sp.: AF46, AF47, AF52 and CC32, *Pseudomonas*: B98C39, B98SK51b, B98SK53b and B98Sk52 and *Pseudoalteromonas* sp. JC29) were able to disrupt the QS activity of two different AHLs, C4 and 3OC8. The remaining three *Pseudoalteromonas* sp. strains (W3, W11 and W21) displayed QSI activity against the three different AHLs tested (C4, C6 and 3OC8).

**Table 4:** Inhibition of QS system by marine sponge bacterial supernatants

Bacterial strains	Quorum quenching activity towards specific AHLs		
	*S. marcescens* SP19	*C. violaceum* CV026	
	C4^a^	C6^b^	3OC8^c^
*Bacillus sp*.			
AF46	+^d^	−e	+
AF47	+	−	+
AF52	−/+^f^	−	+
B9853	−	+	+
CC32	+	−	+
*Paracoccus sp*.			
JM45	−	−	+
*Pseudoalteromonas sp*			
J10	−	−	+
JC29	+	−	+
W3	+	+	+
W11	+	−/+	+
W21	+	−/+	+
*Pseudomonas sp*.			
B98C39	−/+	−	+
B98SK51b	−/+	−	+
B98SK53b	+	−	+
B98SK52	+	−	+
B98SM8	−	−	+
*Psychrobacter sp*.			
B98C22	−	−	+
*Staphylococcus sp*.			
B98C566	−	−	+

**a.** C4: *N*‐Butyryl‐dl‐homoserine lactone.

**b.** C6: *N*‐Hexanoyl‐l‐homoserine lactone.

**c.** 3OC8: *N*‐(3‐Oxooctanoyl)‐l‐homoserine lactone.

**d.** +: AHL inhibition, inhibition of pigment production.

**e.** −: no AHL inhibition, no inhibition of pigment production.

**f.** −/+: weak response of AHL inhibition. Comparison of pigment production inhibition in respect to the control.

To determine if the QSI activity shown by the different marine bacterial supernatants was related with the action of extracellular QQ enzymes (mostly acylases and lactonases), a thermostability assay was performed using the biosensor *C. violaceum* CV026 and 3O8‐HSL, the only AHL whose activity was inhibited by all marine bacterial supernatants (Fig. S3). Heat treatment at 85°C for 1 h was not able to disrupt the QSI activity of the marine supernatants. It is well known that different *Bacillus* species possess a potent QQ activity due to the presence of extracellular enzymes (Lee *et al*., 2002). Therefore, an extra heat treatment of 95°C for 30 min was applied specifically to the marine *Bacillus* supernatants. As before, this extra heat treatment was not able to disarm the QSI activity of the marine *Bacillus* isolate supernatants. Therefore, a small molecule or other inhibitory factor would appear to underpin the QSI activity of the isolates presented in this study.

### Biofilm disruption by marine bacterial supernatants

**Figure 2:**
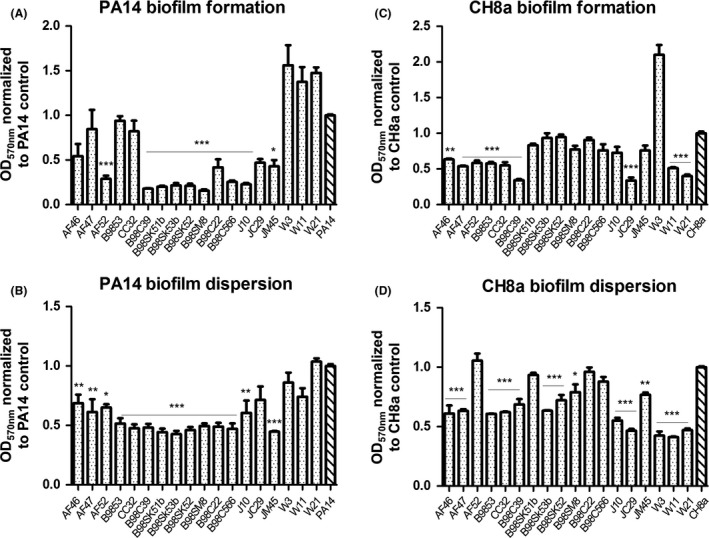
Biofilm formation inhibition and dispersion of *P. aeruginosa* PA14 and *B. subtilis* CH8a by marine bacterial supernatants. A. Biofilm formation inhibition of PA14. B. Biofilm formation inhibition of CH8a. C. PA14 dispersion assay. D. CH8a dispersion assay. Data presented are the mean (±SEM) of at three independent biological replicates and are normalized to the PA14 or CH8a controls. In each case, the individual replicates of the untreated control are normalized to the mean. Statistical analysis was performed using one‐way ANOVA with post hoc Bonferroni testing (*P ≤ 0.05, **P ≤ 0.005, ***P ≤ 0.001).

**Figure 3:**
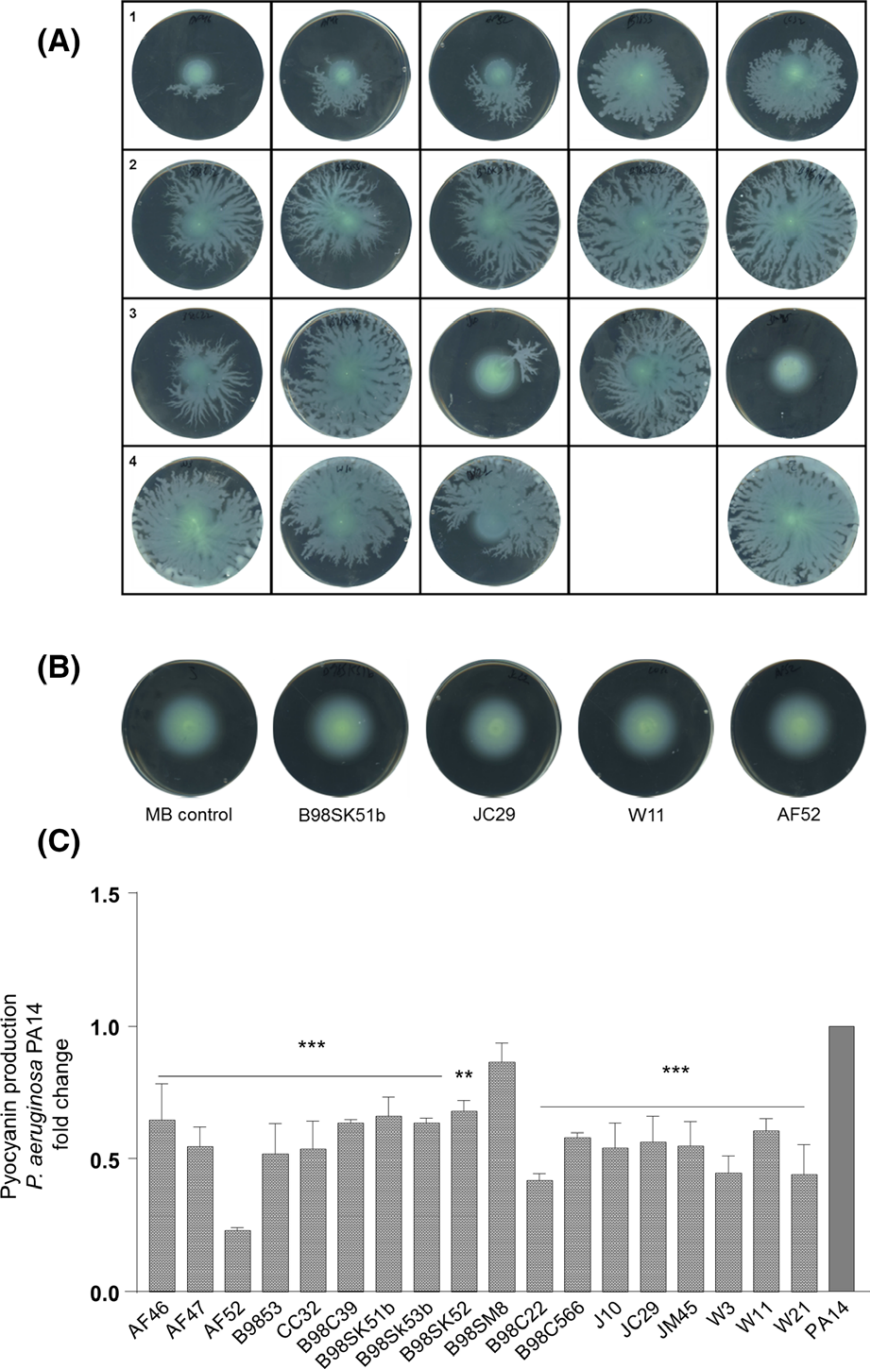
QQ marine bacterial supernatants impair different virulence phenotypes of *P. aeruginosa* PA14. A. Impact of marine bacterial supernatants on PA14 swarming motility. Row 1, from left to right: *Bacillus* sp. AF46, AF47, AF52, B9853 and CC32. Row 2, from left to right: *Pseudomomas* sp. B98C39, B98SK51, B98SK53B, B98SK52 and B98SM8. Row 3, from left to right: *Psychrobacter* sp. B98C22, *Staphylococcus* sp. B98C566, *Pseudoalteromonas* sp J10 and JC29 and *Paracoccus* sp. JM45. Row 4, from left to right: *Pseudoalteromonas* sp. W3, W11 and W21. The untreated MB control is presented on the far right of this row. B. Impact of marine bacterial supernatants on PA14 swimming motility. C. Impact of marine bacterial supernatant on PA14 pyocyanin production. Data presented are normalized to the PA14 control and are the mean (±SEM) of at three independent biological replicates. In each case, the individual replicates of the untreated control are normalized to the mean. Statistical analysis was performed using one‐way ANOVA with post hoc Bonferroni testing (**P ≤ 0.005, ***P ≤ 0.001).

The finding that the QSI activity attributed to the marine sponge strains may be molecular rather than enzymatic, suggests that these compounds could have a potential application for anti‐biofilm development. To test this, the capacity of the marine bacterial strains to either inhibit biofilms or disarm preformed biofilms in the nosocomial pathogen *P. aeruginosa* PA14 and the marine biofouling agent *B. subtilis* CH8a was evaluated (Fig. 3). A wide range of QSI bacterial supernatants significantly impaired the formation of *P. aeruginosa* PA14 biofilms, reducing it by at least 50% (*Bacillus* AF52, the five *Pseudomonas* sp. strains, *Psychrobacter* sp. B98C22, *Staphylococcus* sp. B98C566, *Pseudoalteromonas* sp. J10 and *Paracoccus* sp. JM45) (Fig. 2A). The remaining four *Bacillus* sp. strains were not able to inhibit PA14 biofilm formation, while *Pseudoalteromonas* sp. W3, W11 and W21 increased biofilm formation in this pathogen. Importantly, no inhibition of PA14 growth was observed during biofilm formation in the presence of the different marine QSI supernatants (Fig. S4A). Regarding biofilm formation of *Bacillus subtilis* CH8a (Fig. 2B), a different pattern emerged. *Bacillus* sp. (all five strains), *Pseudomonas* sp. B98C39 and *Pseudoalteromonas sp*. JC29 W11 and W21) all suppressed biofilm formation, by up to 60% in the case of *Pseudomonas* sp. B98C39 and *Pseudoalteromonas* sp. JC29 and W21. *Pseudoalteromonas* sp. W3 strongly increased biofilm formation in CH8a. Planktonic CH8a growth was also affected in the presence of supernatants from the four marine *Bacillus* sp. (AF46, AF47, B9853 and CC32), perhaps explaining the apparent reduction in attached CH8a cells observed for these strains (Fig. S4B).

With respect to the disruption of preformed biofilms, 13 of the 18 QSI marine strains (all *Bacillus* sp, all *Pseudomonas* sp., *Psychrobacter* sp. B98C22, *Staphylococcus* sp. B98C566 and *Paracoccus* sp. JM45) significantly reduced preformed biofilms of PA14 (Fig. 2C). Conversely, *Pseudoalteromonas* sp. JC29, W3, W11 and W21 did not measurably affect preformed PA14 biofilms. In relation to the ability of the marine QSI supernatants to disrupt *B. subtilis* CH8a‐preformed biofilms, 14 of the 18 marine QSI bacteria displayed a significant reduction (Fig. 2D). In this case, the most significant reductions in attached biomass were achieved with the addition of supernatants from *Pseudoalteromonas* sp. JC29, W3, W11 and W21. *Bacillus* sp. AF52, *Pseudomonas* sp. B98SK51b, *Psychrobacter* sp. B98C22 and *Staphylococcus* sp. B98C566 did not disrupt preformed biofilms of CH8a (Fig. 2D).

### *Pseudomonas aeruginosa* PA14 virulence determinants are suppressed by marine bacterial supernatants

*Pseudomonas aeruginosa* is a leading nosocomial pathogen, and it represents a significant clinical challenge associated with morbidity and mortality in chronic disease. As antibiotic‐mediated control is currently under significant threat, new strategies to disarm virulence and persistence‐related behaviours in this pathogen are urgently required. *P. aeruginosa* has a well‐studied QS network that controls its virulence behaviour (Bjarnsholt *et al*., 2010; Jimenez *et al*., 2012), chiefly through two AHL systems, LasRI and RhlRI (Latifi *et al*., 1996; Papenfort and Bassler, 2016). The QSI and anti‐biofilm activity of the marine strains suggest they may interfere with other virulence behaviours in this pathogen, such as QS‐controlled phenazine production and swarming motility (Déziel *et al*., 2004; Caiazza *et al*., 2005). As with biofilm formation, most of the marine QSI supernatants were able to disrupt swarming motility in *P. aeruginosa* to some extent (Fig. 3A). For example, *Bacillus* sp. (AF46, AF47 and AF52), *Pseudoalteromonas* sp. J10 and *Paracoccus* sp. JM45 showed the highest reduction in swarming motility. However, no impact on swimming motility was observed for any of the marine bacterial supernatants tested in this study (Fig. 3B). The marine bacterial supernatants also displayed an inhibition towards the production of pyocyanin by *P. aeruginosa* PA14 (Fig. 3C). Four QSI candidates (*Bacillus* sp. AF52, *Psychrobacter* sp. B98C22 and *Pseudoalteromonas* W3 and W21) elicited a potent reduction in pyocyanin production (over 50%) with respect to the control.

## Discussion

Our understanding of the importance of cell–cell communication among microbes is continuing to expand, as new systems and models are uncovered. While care with terminology must be observed, with distinction required between signals, cues and coercive factors, the paradigm of communication between cells is universal (Fuqua and Greenberg, 2002; Perbal, 2003). A recent review by Hmelo (2017) has summarized the phenomenon of quorum sensing in the marine environment, highlighting the importance of these networks in controlling community dynamics in this largely undiscovered natural ecosystem (Hmelo, 2017). Although QS systems in Gram‐negative bacteria were originally described over 40 years ago in a marine bacterium (Nealson *et al*., 1970), the intrigue of many research groups to decipher the role of AHLs molecules in marine biogeochemistry and ecology is quite recent (Hmelo, 2017). Marine sponges in particular have been described as an untapped reservoir of biodiversity and an increasingly important source of novel bioactives natural products (Blunt *et al*., 2016). Although initially attributed to the sponge itself, it has since emerged that sponge‐associated bacteria play a vital role in the production of these compounds (Thoms and Schupp, 2005; Mehbub *et al*., 2014; Wilson *et al*., 2014). Although QS signalling seems to be quite common in the marine environment (Hmelo, 2017), studies that describe the presence and possible role of AHL–QS system in marine sponge‐associated bacteria are limited (Taylor *et al*., 2004; Mohamed *et al*., 2008; Gardères *et al*., 2012; Zan *et al*., 2012). Equally, there is also a lack of data on the prevalence of marine sponge bacteria that are able to block the signalling capacity of quorum sensing system (Saurav *et al*., 2016). Our study brings new knowledge on the capacity of marine sponge bacteria to quench QS signalling, with the first description of a dual‐acting QS/QSI isolate from a marine sponge. Furthermore, we also describe QSI activity in a marine *Psychrobacter* isolate for the first time. While the discovery of novel QSI small molecules is of interest for the development of next‐generation antimicrobials to treat bacterial infections, the role of QS/QSI in shaping the microbe–sponge symbiotic relationship is also worthy of further investigation.

The bacterial isolates that were discovered to have QSI activity were identified as *Bacillus*,* Staphylococcus*,* Pseudomonas*,* Pseudoalteromonas*,* Paracoccus* and *Psychrobacter*. With the exception of *Psychrobacter*, QSI activities (either enzymatic or small molecule) have been previously described for these organisms. However, studies on isolates associated with the marine environment are limited (Romero *et al*., 2011; Yu *et al*., 2013; Saurav *et al*., 2016). Interestingly, as far as we know, this is the first description of a marine sponge *Psychrobacter* sp. strain that can interfere with QS in a model organism, thus broadening the potential for QS signalling among marine microbes. Although three genera accounted for 77% of the QSI activities observed, each isolate included in the study was at least phenotypically distinct (Table 2), a result that was confirmed when activity against specific AHLs was investigated (Table 4). While all marine supernatants inhibited 3OC8‐HSL, only a few of them were able to block C4 and C6‐HSL. We consider two possibilities that may underpin this specificity: (i) the presence of the OH group at the C3 position in the 3OC8‐HSL or (ii) that 3OC8‐HSL molecules are more abundant than other AHLs in the marine sponge environment, and thus select for a higher abundance of antagonist QSIm's. Furthermore, several *Bacillus* strains exhibited antibacterial activity against a panel of clinically relevant isolates suggesting that their QSI activity is only one mechanism used within the ordered symbiotic community of the marine sponge. Antimicrobial activity has been recently reported for other marine sponge *Bacillus* isolates (Matobole *et al*., 2017). In addition, two of the QSI isolates (*Pseudoalteromonas* sp. J10 and *Paracoccus* sp. JM45) also displayed QS activity, being capable of activating the *A. tumefaciens* NTL4 long‐chain biosensor. This dual activity has not been previously described in marine sponge isolates and adds another layer of complexity to the role of QS/QSI in shaping the population dynamics of this ecosystem.

Consistent with their ability to disrupt QS signalling in the biosensor strains, supernatants from the marine QSI strains were able to inhibit biofilm formation and preformed biofilms, (Fig. 2A and B) respectively, swarming motility (Fig. 3A) and pyocyanin production (Fig. 3C) at different levels (all data summarized in Fig. S5). Interestingly, swimming motility was unaffected in the presence of QSI supernatants, perhaps a reflection of the fact that it is not considered a multicellular behaviour (Kearns, 2010). There appears to be a genera‐specific signature to the QSI activity whereby *Pseudoalteromonas sp*. did not inhibit *P. aeruginosa* biofilm PA14 formation, but were quite active against *B. subtilis* CH8a. Conversely, *Pseudomonas sp*. inhibited PA14 biofilm formation, while it did not significantly reduce biofilm formation in CH8a. Future isolation and characterization of the active fraction underpinning the QSI activity will provide answers as to what governs this pattern of activity, with genome sequencing being required to unravel the molecular pathways involved in QSIm production.

## Experimental procedures

### Bacterial strains and culture conditions

The marine bacterial strains analysed in this study are summarized in Table 2. Marine agar or broth (MA or MB respectively) was routinely used to grow marine bacterial strains (Difco, Oxford, UK). SYP agar with 1.5% of sea salt and MA or MB were used in different experiments performed in this work to analyse the potential QSI capability of marine bacteria. All marine bacterial strains used in this study were grown routinely at 23°C. Different *N*‐Acyl homoserine lactones (AHLs) used in this work were purchased from Sigma‐Aldrich (Cambridge, UK) and were dissolved in DMSO (*N*‐butyryl‐dl‐homoserine lactone: C4‐HSL; *N*‐hexanoyl‐l‐homoserine lactone: C6‐HSL; *N*‐(3‐oxooctanoyl)‐l‐homoserine lactone: 3OC8‐HSL; *N*‐(3‐oxodecanoyl)‐l‐homoserine lactone: 3OC10‐HSL; and *N*‐(3‐oxododecanoyl)‐l‐homoserine lactone: 3OC12‐HSL).

### Isolation and characterization of potential QSI marine isolates

A screening pipeline to mine QS antagonists from a collection of culturable bacteria isolated from marine sponge samples was designed (Fig. S1). A total of 440 marine bacterial isolates were previously selected from different marine sponge samples to present different colony morphologies and pigmentation on MA plates (MA) (Difco, Oxford, UK). These bacterial isolates were collected from different sponges belonging to different families such as Hexactinellida, Polymastia, Cliona, Haliclonas, Stelleta, Lissodendoryx, Poecillastra, Inflatella and Axinella. These sponge samples were collected using a remote‐operated vehicle on board the Celtic Explorer research vessel, 300 nautical miles off the west coast of Ireland as part of the marine biodiscovery cruise carried out in May 2010. These bacterial isolates were initially tested for their potential QSI activity against three different biosensor reporter strains. From this original screening, 18 bacterial isolates were selected as possible QSI candidates (Table 2).

### Phylogenetic analysis

Genomic DNA from 18 bacterial isolates was extracted using UltraClean^®^ Microbial DNA Isolation Kit (MO BIO, USA) following the manufacturer guidelines. PCR based on the amplification of 16S rRNA using the universal primers 27F (5′‐AGAGTTTGATCMTGGCTCAG‐3′) and 1492R (5′‐TACGGYTACCTTGTTACGACTT‐3′) was performed (da Silva *et al*., 2013, Lane, 1991). PCR amplicons were sequenced by MWG Eurofins, UK. The 16S rRNA sequences were assembled and edited with the software Vector NTI Suite 9, and then were compared with those available in NCBI database through BLASTn searches (Altschul *et al*., 1997). For the phylogenetic analysis, three of the closest sequences for each sequence obtained in this study were taken from Genbank, and subsequently, a multi‐alignment was carried out using Clustal Omega (http://www.ebi.ac.uk/Tools/msa/clustalo/). A maximum‐likelihood phylogenetic tree was constructed by Mega 6.06 (Tamura *et al*., 2013) using the Kimura two‐parameter model and the option of complete deletion to eliminate positions containing gaps. Confidence levels of the branching points were determined using 100 bootstrap replicates.

### Quorum sensing inhibition biosensor assay

The following biosensor reporter strains were used to identify the QSI ability of the different marine bacterial isolates: *Serratia marcescens* SP15 for the detection of QSI activity against short‐chain AHLs (Poulter *et al*., 2010), *Chromobacterium violaceum* DSM 30191 for the detection of QSI activity against medium‐chain AHLs (Morohoshi *et al*., 2008) and *Agrobacterium tumefaciens* NTL4 for the detection of QSI activity against long‐chain AHLs (*A. tumefaciens* NTL4 contains the plasmid pZLR4 carrying a *traG*::*lacZ* reporter fusion (Farrand *et al*., 1996; Yin *et al*., 2012a, b). Whole cells of marine bacterial strains were cultured on MA and SYP‐1.5 for 72 h at 23°C. Then, they were overlaid with LB soft agar (0.5% agar) inoculated with SP15 and DSM 30191 QSI biosensors strains at an OD 600 nm of 0.25. *A. tumefaciens* NTL4 was also inoculated at an OD600 nm of 0.25 but in MM minimal medium (K_2_HPO_4_: 10.5 g, KH_2_PO_4_: 4.5 g, MgSO_4_·7H_2_O: 0.2 g, calcium dehydrate: 0.01 g, (NH_4_)_2_SO_4_: 2 g, FeSO_4_: 0.005 g, MnCl_2_·4H_2_O, d‐Mannitol: 2 g and agarose: 5 g) supplemented with 50 μg ml^−1^ of 5‐bromo‐4chloro‐3‐indolyl‐β‐d‐galactopyranoside and 20 nM of 3OC10 and 3OC12. QSI activity was identified by a colour inhibition halo around the growth of each marine bacterial strain.

### Generation of marine bacterial supernatants

One hundred millilitre of MB were inoculated from overnight MB fresh cultures of each marine bacterium at an OD600 nm of 0.02 and were incubated for 72 h at 23°C at 170 r.p.m.. Then, bacteria were removed by centrifugation and the supernatants were filter sterilized using a Sartorius AG vacuum filtration system (0.22 μm).

### QSI activity of marine bacterial supernatants towards specific AHLs

To decipher the range of specific quorum quenching activity of each marine bacterial strain, an experimental approach was performed as described previously (Gutierrez‐Barranquero *et al*., 2015) with minor modifications. 15 ml of LB agar 1% mixed with 5 ml of supernatant from each marine bacterial strain were used to prepare the agar plates. Then, prior to pouring LB‐supernatant agar plates, the biosensors *S. marcescens* SP19 and *C. violaceum* CV026 were inoculated at an OD 600 nm of 0.25. Subsequently, wells were made in the LB‐supernatant agar plates, and 20 μl of a 50 μM concentration of different AHLs and 20 μl of DMSO as control were added. DMSO was the solvent where the different AHLs were previously dissolved. C4‐HSL was used for the plates inoculated with *S. marcescens* SP19, and C6‐HSL and 3OC8‐HSL for the plates inoculated with *C. violaceum* CV026. LB 1% (15 ml) mixed with 5 ml of marine broth and also inoculated with the different biosensors were used as control plates. Finally, plates were incubated for 24 h at 30°C. QSI activity was identified by pigment inhibition around the well where the different AHLs were inoculated, respect to the control plates.

### Thermostability of marine bacterial supernatants

To stablish the thermostability of the putative QQ enzymes or QSIm in the different marine bacterial supernatants, supernatant from each one was heated to 85°C for 1 h. The supernatants of the five *Bacillus* strains included in this study were also subjected to an additional heat treatment of 95°C for 30 mins. After these heat treatments, the different supernatants were used to prepare LB‐supernatant agar plates inoculated with *C. violaceum* CV026 as described above. Wells were made in the LB‐supernatant agar plates, and 20 μl of a 50 μM concentration of 3OC8‐HSL was added. Supernatants not heat treated, and also, MB heat and not heat treated were used as control.

### Biofilm formation of *Pseudomonas aeruginosa* PA14 and *Bacillus subtilis* CH8a

Biofilm formation experiments with minor modifications were carried out as previously described (O'Toole and Kolter, 1998). The opportunistic human pathogen *P. aeruginosa* PA14 and the biofouling agent *Bacillus subtilis* CH8 were used to analyse the anti‐biofilm activity of the marine bacterial supernatants. PA14 and CH8a from a fresh overnight culture (LB broth and MB respectively) were used to inoculate 500 μl of LB broth and MB, mixed with 500 μl of the different marine bacterial supernatant in 24‐well microtitre plates (Starstedt, UK) at an OD600 nm of 0.05. *P. aeruginosa* PA14 biofilms were incubated at 37°C for 24 h and *Bacillus subtilis* CH8a biofilms were incubated at 23°C for 72 h, both statically. Biofilm formation was measured by solubilization of crystal violet with 96% ethanol and quantified at 570 nm.

### Dispersion assay of *P. aeruginosa* PA14 and *Bacillus subtilis* CH8a

The capability to inhibit preformed biofilm of *P. aeruginosa* PA14 and *B. subtilis* CH8a by the different marine bacterial supernatants was analysed. Fresh LB and MB overnight culture of *P. aeruginosa* PA14 and *B. subtilis* CH8a, respectively, were used to inoculate 150 μl of LB and MB at an OD600 nm of 0.05 in 96‐well microtitre plates (Sarstedt, Leicester, UK). Biofilms then were incubated at 37°C for 24 h for *P. aeruginosa* PA14 and 23°C for 72 h for *B. subtilis* CH8a, both statically. After this time of incubation, 150 μl of the different marine bacterial supernatants were added to the wells where the biofilms were developed. The preformed biofilm mixed with the marine bacterial supernatants were incubated for another 24 h at 37°C and for another 72 h at 23°C for *P. aeruginosa* PA14 and *B. subtilis* CH8a respectively. 150 μl of MB was used as positive control. Preformed biofilms inhibition was measured by solubilization of crystal violet with 96% ethanol and quantified at 570 nm.

### PA14 swarming and swimming motility

Swarming and swimming motilities of *P. aeruginosa* PA14 were tested in the presence of the different marine bacterial supernatants. For swarming plates, 15 ml of Eiken‐swarming agar (0.8% Eiken nutrient broth, 0.6% Eiken agar and 0.5% glucose) were mixed with 5 ml of MB supernatant. For swimming plates, 15 ml LB agar 0.3% were mixed with 5 ml of MB supernatants The bacterial cells of *P. aeruginosa* PA14 were gently inoculated using a toothpick at the centre of the agar surface, and the plates were incubated at 37°C for 18 and 24 h for swarming and swimming respectively.

### PA14 pyocyanin production

Overnight cultures of *P. aeruginosa* PA14 were inoculated into 2.5 ml of LB broth + 2.5 ml of the different marine bacterial supernatants at an OD600 nm of 0.05. PA14 cultures were then incubated at 37°C for 24 h at 180 r.p.m. The total volume of the culture was centrifuged at 3320 × g for 10 min. Chloroform (3 ml) was added to the supernatant fraction and vortexed. The bottom phase was then recovered following centrifugation at 3320 × g for 5 min, and 2 ml of 0.2 M HCl was added. The samples were then vortexed for 20 s, allowed to settle, and the absorbance of the pink layer upper phase was measured at 570 nm.

### Quorum sensing screening assay

The following reporter strains were used to identify AHL production by the different marine bacterial strains: *S. marcescens* SP19 (Poulter *et al*., 2010) was used to detect the production of short‐chain AHLs; *C. violaceum* CV026 (McClean *et al*., 1997) was used to detect medium‐chain AHLs and *A. tumefaciens* NTL4 was used for the detection of long‐chain AHLs. Marine bacterial strains were cultured on MA and SYP‐1.5 for 72 h at 23°C. Then, they were overlaid with LB soft agar (0.5% agar) inoculated with the different biosensors strains at an OD 600 nm of 0.25, with the exception that for *A. tumefaciens* NTL4 a MM was used and supplemented with 50 μg ml^−1^ of 5‐bromo‐4chloro‐3‐indolyl‐β‐d‐galactopyranoside. Overlaid plates were incubated for 24 h at 30°C. Quorum sensing activity was identified by prodigiosin (red colour) due to the production of short‐chain AHLs, violacein (purple colour) by the production of medium‐chain AHLs and blue colour production by the breakdown of 5‐bromo‐4‐chloro‐3‐indolyl‐β‐d‐galactopyranoside by the production of long‐chain AHLs.

### Antimicrobial plate overlay assay

Whole cells of marine bacterial strains were cultured on MA plates for 72 h at 23°C. The different bacterial indicator strains were grown overnight in LB broth. Then, they were inoculated at an OD600 nm of 0.1 in LB soft agar (0.5%) and overlaid the marine bacterial strains. The fish pathogens *V. anguillarum* and *E. tarda*, an environmental hospital isolate *E. coli* MUH76317, food‐borne pathogen *S. typhimurium* C5369 and the opportunistic human pathogens *P. aeruginosa* PA14, *B. cepacia* NCTC10743 and *S. aureus* NCDO949 were used to test the potential antimicrobial activity of marine bacterial strains. Zones of inhibition were tested after 24 h of incubation at 37°C.

### Statistical analysis

Data analysis was performed using GraphPad Prism 5.03 (San Diego, CA, USA). At least three independent biological replicates were performed for all experiments. Statistical analyses were performed using one‐way ANOVA with Bonferroni post hoc testing, or two‐tailed paired Student's *t*‐test. Differences were considered significant when the *P* value was ≤ 0.05 (*P ≤ 0.05, **P ≤ 0.005).

### Nucleotide sequence accession numbers

The partial 16S rRNA sequences of the different marine sponge bacteria used in this study were deposited in GenBank (NCBI) under the accession numbers: MF289535‐MF289552.

## Conflict of Interest

The authors declare there is no conflict of interest in the submission of this manuscript.

## Supporting information


**Fig. S1.** Schematic representation of the screening pipeline protocol used to decipher the QQ potential of marine isolates.

**Fig. S2.** QS activation of the *A. tumefaciens* NTL4 biosensor by two marine bacteria, *Pseudoalteromonas* sp. J10 (up picture) and *Paracoccus* sp. JM45 (down picture).

**Fig. S3.** Thermostability of QQ marine bacterial supernatants. (A) Non‐heat treated plates. (B) Heat – treated plates.

**Fig. S4.** Biofilm (OD_600 nm_) of (A) *P. aeruginosa* PA14 and (B) *B. subtilis* CH8a.

**Fig. S5.** Suppression of primary virulence phenotypes regulated by QS in *P. aeruginosa* by marine sponge QQ isolates. Red colour: no inhibition of the virulence phenotype. Green light colour: inhibition of the virulence phenotype ≤ 50%. Green dark colour: inhibition of the virulence phenotype > 50%. Yellow colour: Promotes the increased of the virulence phenotype.
