# Supplementary figures and images for: Disruption of N‐acyl‐homoserine lactone‐specific signalling and virulence in clinical pathogens by marine sponge bacteria
Source: Microb Biotechnol. 2017 Nov 3;12(5):1049–63. doi: 10.1111/1751-7915.12867 (PMC6680641; doi:10.1111/1751-7915.12867)

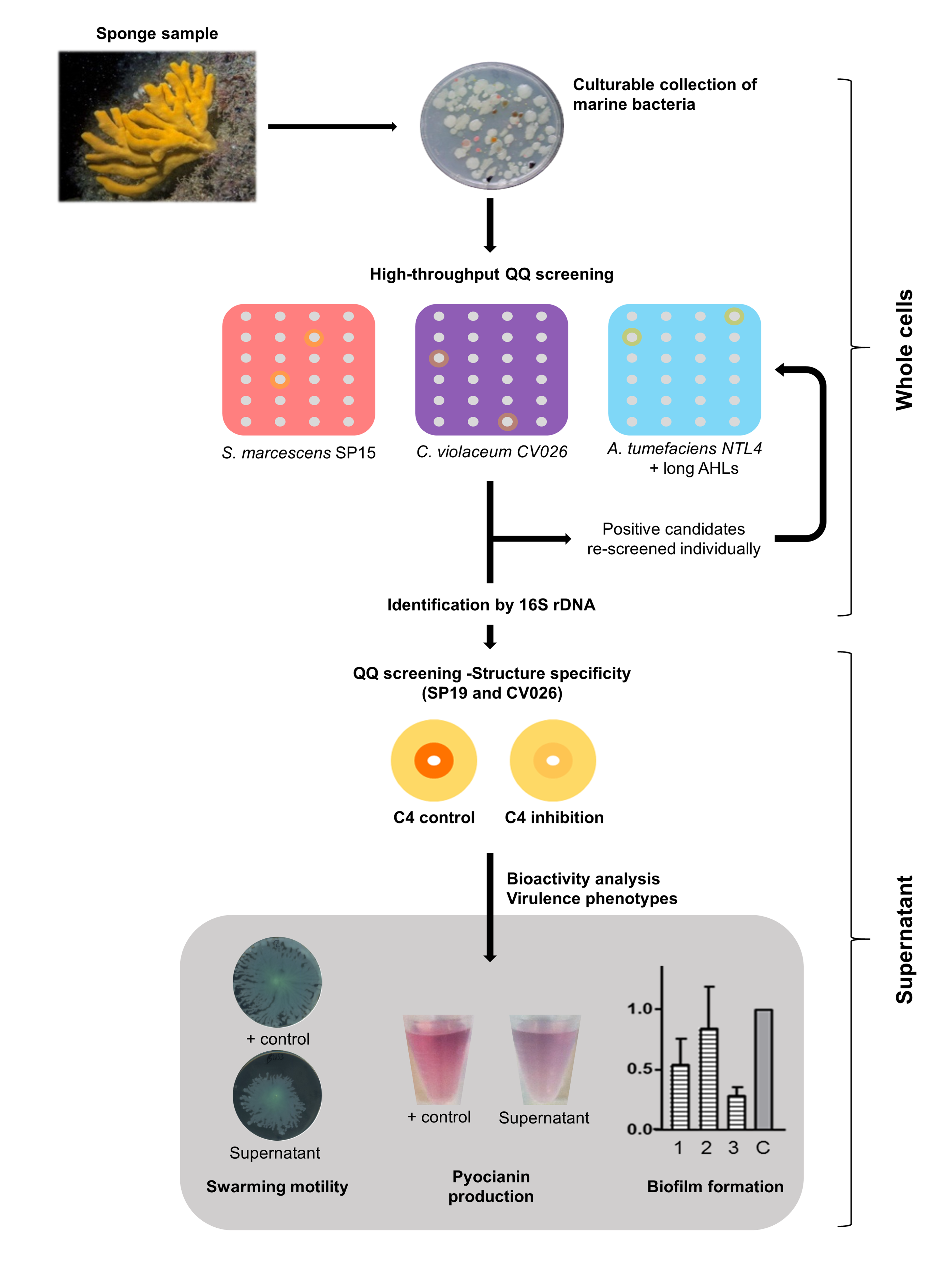

Supplement: Supplementary file 1 — Fig. S1. Schematic representation of the screening pipeline protocol used to decipher the QQ potential of marine isolates. [file MBT2-12-1049-s001.tif]

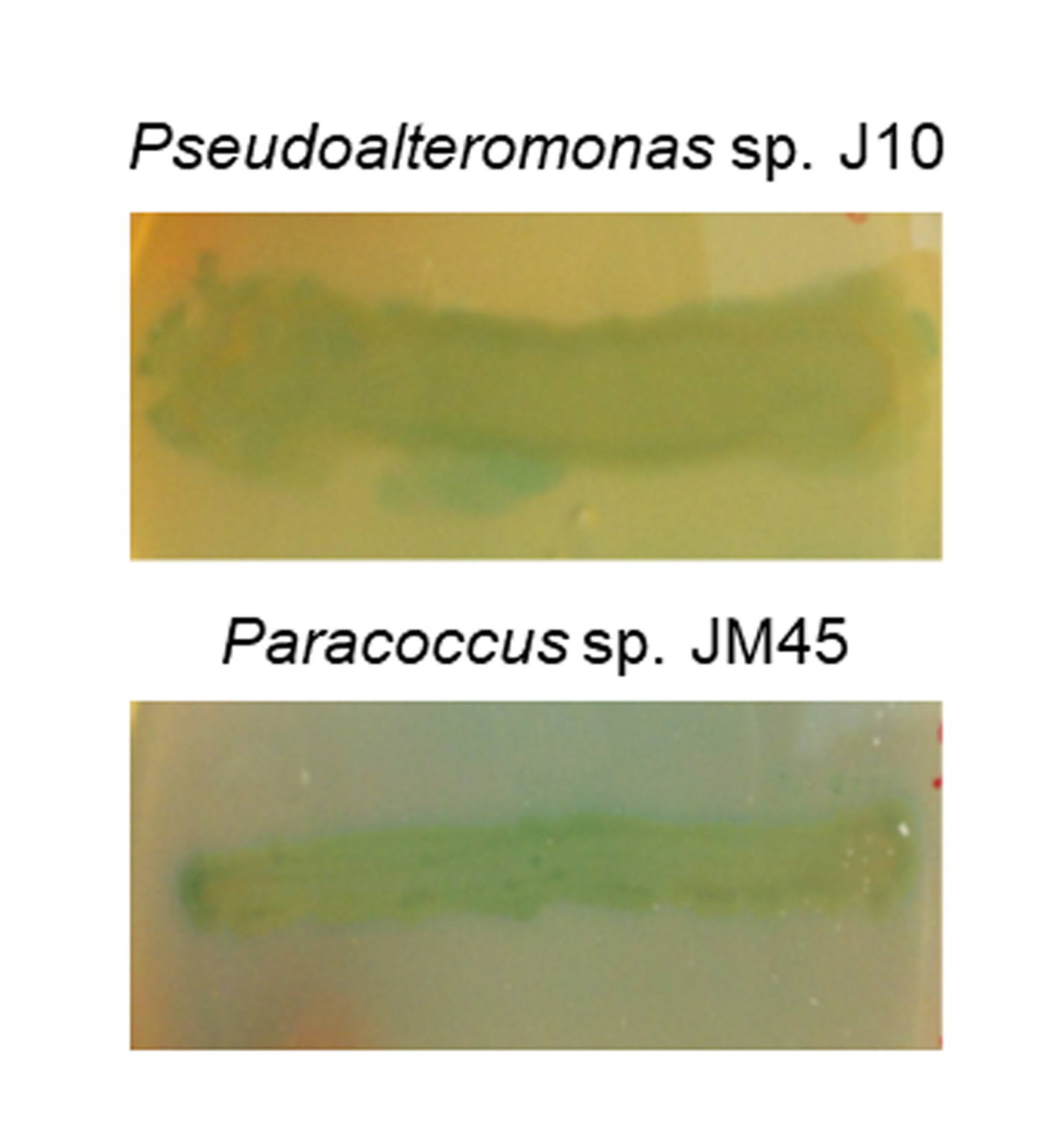

Supplement: Supplementary file 2 — Fig. S2. QS activation of the A. tumefaciens NTL4 biosensor by two marine bacteria, Pseudoalteromonas sp. J10 (up picture) and Paracoccus sp. JM45 (down picture). [file MBT2-12-1049-s002.tif]

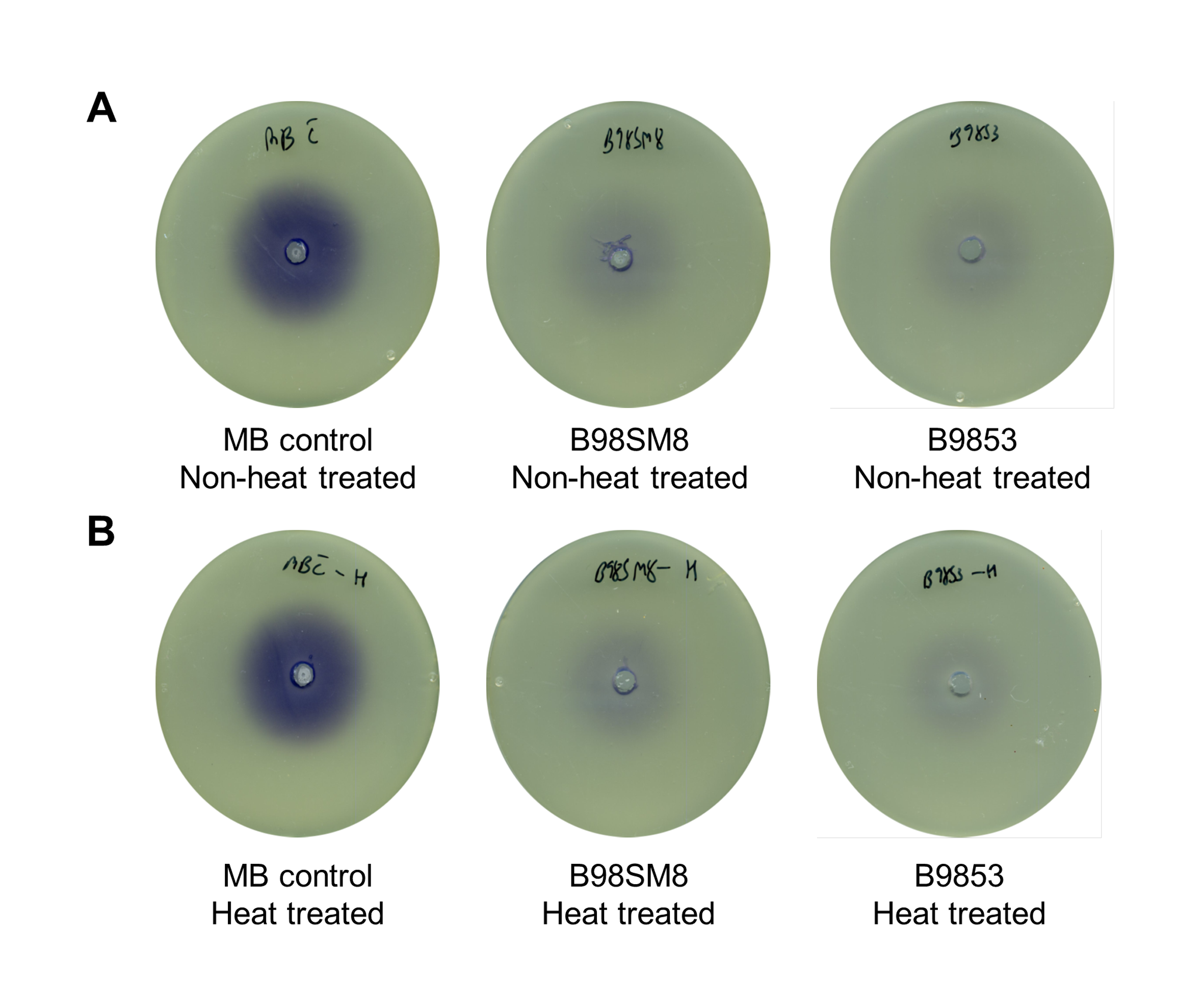

Supplement: Supplementary file 3 — Fig. S3. Thermostability of QQ marine bacterial supernatants. (A) Non‐heat treated plates. (B) Heat – treated plates. [file MBT2-12-1049-s003.tif]

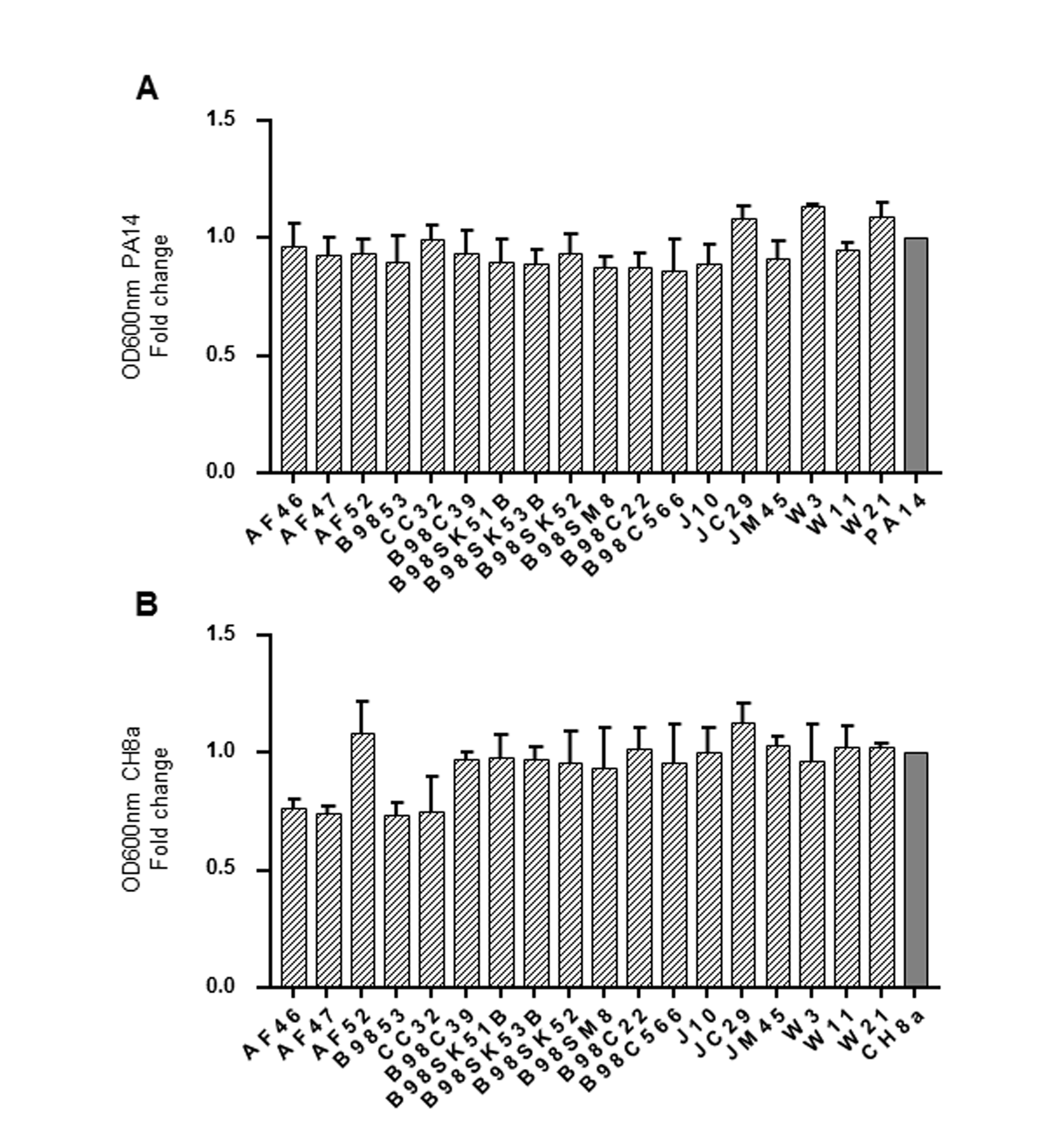

Supplement: Supplementary file 4 — Fig. S4. Biofilm (OD600 nm) of (A) P. aeruginosa PA14 and (B) B. subtilis CH8a. [file MBT2-12-1049-s004.tif]

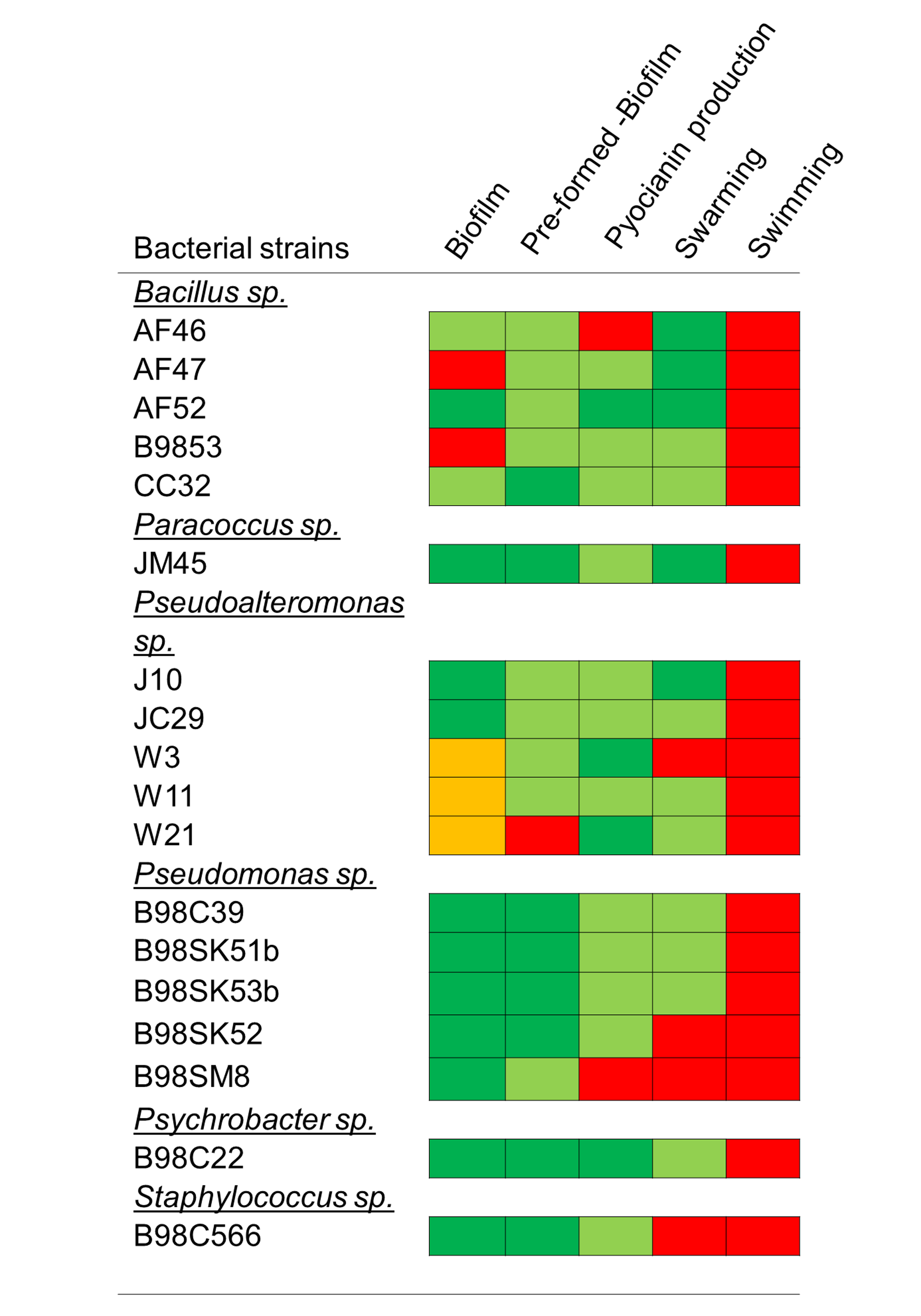

Supplement: Supplementary file 5 — Fig. S5. Suppression of primary virulence phenotypes regulated by QS in P. aeruginosa by marine sponge QQ isolates. Red colour: no inhibition of the virulence phenotype. Green light colour: inhibition of the virulence phenotype ≤ 50%. Green dark colour: inhibition of the virulence phenotype > 50%. Yellow colour: Promotes the increased of the virulence phenotype. [file MBT2-12-1049-s005.tif]
