# Supplementary material for: Disruption of N‐acyl‐homoserine lactone‐specific signalling and virulence in clinical pathogens by marine sponge bacteria
Source: Microb Biotechnol. 2017 Nov 3;12(5):1049–63. doi: 10.1111/1751-7915.12867 (PMC6680641; doi:10.1111/1751-7915.12867)
Supplement: Supplementary file 6 [file MBT2-12-1049-s006.docx]

**Supporting information**

**Fig. S1.** Schematic representation of the screening pipeline protocol used to decipher the QQ potential of marine isolates.

**Fig. S2.** QS activation of the *A. tumefaciens* NTL4 biosensor by two marine bacteria, *Pseudoalteromonas* sp. J10 (up picture) and *Paracoccus* sp. JM45 (down picture).

**Fig. S3.** Thermostability of QQ marine bacterial supernatants. (A) Non-heat treated plates. (B) Heat- treated plates.

**Fig. S4.** Biofilm (OD_600nm_) of (A) *P. aeruginosa* PA14 and (B) *B. subtilis* CH8a.

**Fig. S5.** Suppression of primary virulence phenotypes regulated by QS in *P. aeruginosa* by marine sponge QQ isolates. Red colour: no inhibition of the virulence phenotype. Green light colour: inhibition of the virulence phenotype ≤ 50%. Green dark colour: inhibition of the virulence phenotype > 50%. Yellow colour: Promotes the increased of the virulence phenotype.
